# Complete Response to Nivolumab and Ipilimumab After Pazopanib Discontinuation in a Seven‐Year‐Old Girl With Alveolar Soft Part Sarcoma: A Case Report and Literature Review

**DOI:** 10.1002/cnr2.70305

**Published:** 2025-08-16

**Authors:** Aziz Eghbali, Mobin Obeidinia, Hamideh Sadat Mirmohammadi, Roghayeh Rahimiafazal, Arya Shirani

**Affiliations:** ^1^ Clinical Research Development Center of Aliasghar Hospital Iran University of Medical Sciences Tehran Iran; ^2^ Research Scholar, Gastrointestinal and Liver Diseases Research Center, Firoozgar General Hospital Iran University of Medical Sciences Tehran Iran; ^3^ Department of Pediatric Hematology and Oncology Shahid Beheshti University of Medical Sciences, Mofid Children Hospital Tehran Iran; ^4^ Cardiology Resident, School of Medicine Tehran University of Medical Sciences Tehran Iran

**Keywords:** alveolar soft part sarcoma, immune checkpoint inhibitor, ipilimumab, nivolumab, pazopanib, tyrosine kinase inhibitor

## Abstract

**Background:**

Alveolar soft part sarcoma (ASPS) is an extremely rare neoplasm that often presents with metastatic disease at diagnosis, leading to a poor prognosis. Conventional chemotherapy is generally ineffective against ASPS. Recent studies, nonetheless, suggest that tyrosine kinase inhibitors (TKIs) and immune checkpoint inhibitors (ICIs) may be effective treatment options.

**Case:**

We present a seven‐year‐old girl with stage IV ASPS in whom initial therapy with pazopanib, a TKI, had resulted in significant gastrointestinal side effects and poor drug compliance. We switched her treatment to an ICI regimen, a combination of nivolumab and ipilimumab, which led to a complete response.

**Conclusion:**

This case highlights the value of ICIs as potential treatment options for ASPS in the pediatric population, especially in patients who cannot tolerate TKIs' side effects.

**Abbreviations**
anti‐PD‐L1: anti‐programmed cell death ligand‐1
ASPS: Alveolar soft part sarcoma
CNB: core needle biopsy
CR: complete response
CSCO: Chinese Society of Clinical Oncologists
CTLA‐4: Cytotoxic T‐lymphocyte‐associated protein 4
FDG PET/CT: fluorodeoxyglucose‐positron emission computed tomography
GI: gastrointestinal
NCCN: National Comprehensive Cancer Network
PD‐1: programmed cell death protein‐1
SD: stable disease
TKI: Tyrosine kinase inhibitor

## 1 Introduction

Alveolar soft part sarcoma (ASPS) is an ultra‐rare malignancy accounting for < 1% of all soft tissue sarcomas [1]. ASPS typically occurs in young individuals ranging from 15 to 35 years old. While unusual sites have been reported for the primary tumor, the lower extremities for adults and the head and neck region for children remain the typical locations for developing ASPS [2]. However radiologic studies can help with the diagnosis of ASPS, definite diagnosis is dependent mainly on its characteristic histopathologic features and a specific translocation, der (17)t(X;17) (p11.2; q25), leading to ASPSCR1–TFE3 gene fusion protein [3]. Microscopically, ASPS is identified by its distinct pattern of uniform cells with or without multinucleation, put into nest‐like clusters separated by fibrovascular septa, and the existence of periodic acid Schiff components [4].

Despite its slow growth pattern, ASPS has a poor prognosis due to its high metastasis potential, with the most common sites being the lungs, central nervous system, and bones [2]. The 5‐year survival rate is reported to be 20%–65% at diagnosis, possibly higher in the pediatric population [3, 5].

As a significant proportion of patients diagnosed with ASPS already have metastatic disease, management plans are primarily medical rather than surgical [6]. ASPS is generally considered resistant to conventional cytotoxic chemotherapeutic drugs, and chemotherapy agents play no role in its management [7]. While some studies in recent years have supported the use of tyrosine kinase inhibitors (TKIs), such as pazopanib and nivolumab, along with with ipilimumab, an immune checkpoint inhibitors (ICI) in the management of ASPS, these recommendations are based on limited and controversial available evidence up to now [2, 8]. ICIs, like nivolumab, are reserved for patients with ASPS who have failed to be treated with other therapies (e.g., pazopanib) and have shown inconsistent results in reported cases, ranging from disease progression to promising outcomes [6, 9]. Thus, more studies need to be conducted to provide evidence in support of these therapeutic options for ASPS regarding the scarcity of data and lack of affirmation in the pediatric population [10]. In this case report, we present a rare case of a child with ASPS who experienced adverse effects from pazopanib. The patient showed a complete response after switching to the ICI combination of nivolumab and ipilimumab.

## 2 Case Presentation

This study reports the clinical course of a previously healthy 5‐year‐old girl brought to the pediatric oncology clinic, Mahak Children's Hospital, Tehran, Iran, in February 2022 with a 4 × 4 cm firm, non‐tender, slowly growing mass located in the left paramedian sublingual region for 6 months. A core‐needle biopsy (CNB) of the mass revealed nodules of eosinophilic large cells with small vesicular nuclei, highly suggestive of ASPS (Table 1).

**TABLE 1:** Timeline of the patient's clinical course.

Timeline	Clinical manifestation	Imaging finding	Intervention
August 2021	Emerging mass in the sublingual region	No imaging has been performed yet	No intervention implemented
February 2022	The mass reached its maximum size	MRI revealed a T1 hypointense signal, a well‐defined mass with heterogeneous enhancement	The patient underwent CNB of the mass
March 2022	Total surgical excision of the mass	FDG PET/CT showed multiple pulmonary nodules (Figure 1)	Initiation of pazopanib based on diagnosis of stage IV ASPS
June 2022	The patient complained about GI adverse effects, suggesting the pazopanib side effects	Chest CT scan showed multiple pulmonary nodules (Figure 2a,b)	Pazopanib continued
December 2022	Poor compliance due to deterioration of GI side effects and hair depigmentation	Chest CT scan revealed stable pulmonary nodules (Figure 2c)	Switching pazopanib to an ICI combination regimen (nivolumab concurrent with ipilimumab)
July 2023	The patient's compliance improved	Chest CT scan indicating vanishing of pulmonary nodules in favor of CR (Figure 2d)	The ICI regimen continued
March 2024	Well compliance with treatment and improvement in general condition	CT scan indicating CR	Termination of the treatment, and the patient has been advised to schedule regular follow‐up appointments
December 2024	The latest follow‐up visit revealed an asymptomatic patient with no complaints and no signs of disease recurrence	None	Follow‐up visit 6 months later

Abbreviations: ASPS, alveolar soft part sarcoma; CNB, core‐needle biopsy; CR, complete response; FDG PET/CT, fluorodeoxyglucose‐positron emission computed tomography; ICI, immune checkpoint inhibitor; MRI, magnetic resonance imaging.

Subsequently, the patient underwent total surgical excision of the mass, confirming the diagnosis by a positive reaction for TFE‐3. At the time, the mitotic activity score was 1, with no necrosis, lymphovascular, or perineural invasion.

**FIGURE 1:**
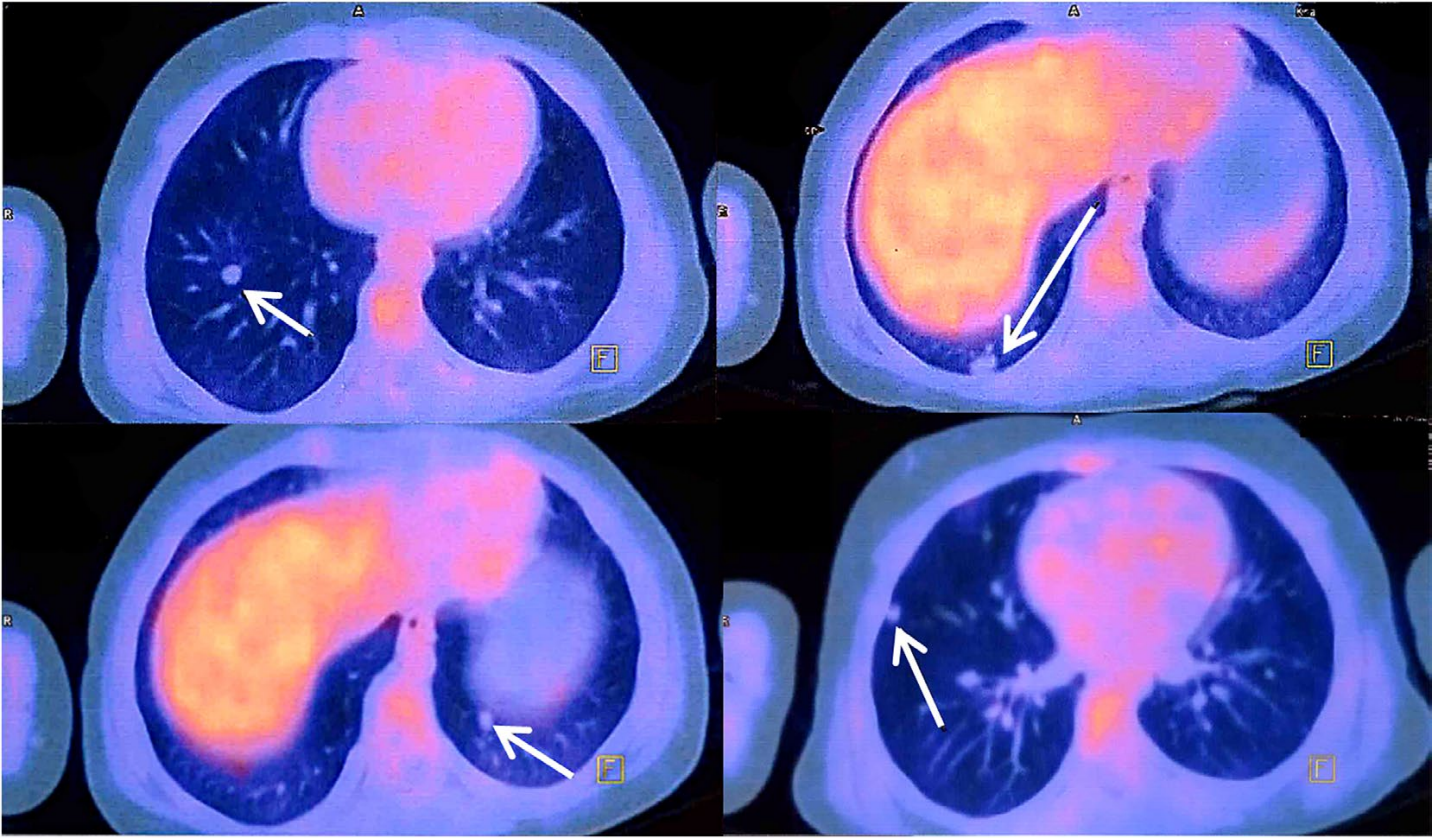
Performed FDG PET/CT in March 2022, showing multiple bilateral pulmonary nodules (arrows) highly concerning for metastatic disease.

**FIGURE 2:**
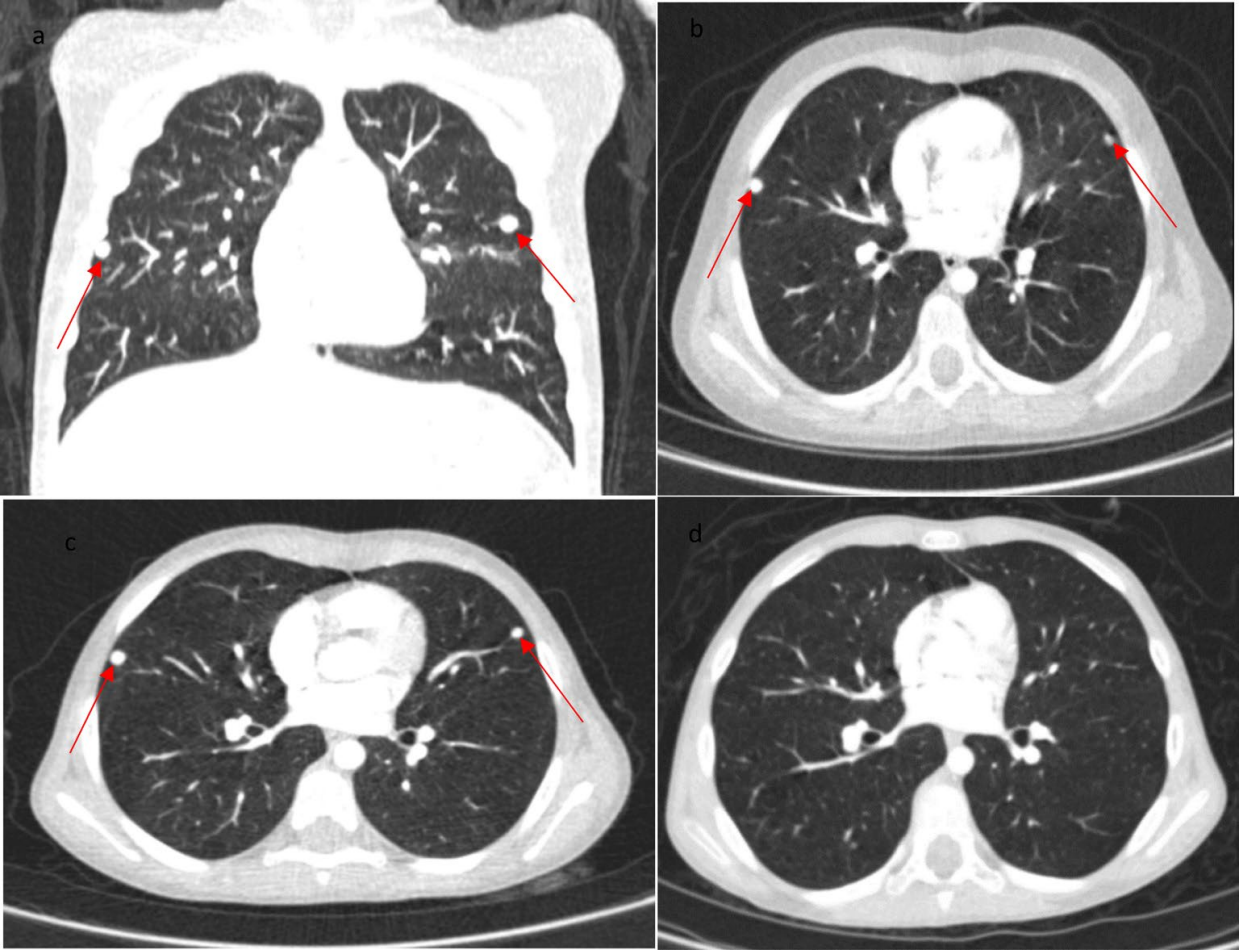
Contrast‐enhanced CT scan of the patient in June 2022, 3 months after initiating pazopanib, demonstrated bilateral multiple pulmonary nodules in coronal (a) and axial views (b) (arrows). CT scan was performed in February 2023, 6 months after initiating pazopanib, showing stable pulmonary nodules (c) (arrows). CT scan of the patient in July 2024, 6 months after initiation of ICI therapy, revealed no pulmonary nodule (d).

To determine the tumor stage, the patient underwent a whole‐body fluorodeoxyglucose‐positron emission computed tomography (FDG PET/CT), revealing multiple small bilateral pulmonary nodules with the greatest diameter of 9 mm (Figure 1). No metabolic evidence of malignancy was detected in other parts of the body. Hence, the patient, diagnosed with stage IV ASPS, started to receive pazopanib, a TKI, in March 2022 at Mahak Children's Hospital. A follow‐up chest CT scan was conducted 3 months later, revealing pulmonary nodules without any signs of improvement or progression (Figure 2a,b). Meanwhile, the patient reported gastrointestinal (GI) side effects, including nausea, vomiting, and loss of appetite, which were most likely side effects of pazopanib. Despite these adverse effects, treatment with pazopanib was continued, and the patient experienced stable disease (SD). The pulmonary nodules were stable on the follow‐up CT scans performed in December 2022 (Figure 2c), when adverse effects of pazopanib deteriorated, and hair depigmentation occurred, resulting in much lower drug compliance. Given the circumstances, we had to revise the treatment plan to a new regimen.

Subsequently, pazopanib was withheld, and the patient underwent nivolumab 1 mg per kilogram (mg/kg) followed by ipilimumab 3 mg/kg for 4 cycles with intervals of 3 weeks. After switching the treatment regimen, the patient's adherence improved, and the pulmonary nodules disappeared in the CT scan conducted 6 months later, showing no signs of metastasis to other parts of the body (Figure 2d). The decreased side effects also contributed to an improved quality of life for the patient.

Subsequently, nivolumab immunotherapy was maintained for an additional year. At the most recent follow‐up appointment in December 2024, the patient remained asymptomatic, had no reported adverse effects, and exhibited no signs of disease recurrence.

## 3 Discussion

We presented a 5‐year‐old girl diagnosed with ASPS who was in SD with pazopanib but could not tolerate it due to GI side effects. Her compliance improved after switching to nivolumab and ipilimumab combination and a complete response to the therapy was observed.

ASPS, a rare sarcoma with a high mortality and morbidity rate, usually presents as a slowly growing, non‐tender mass. This indolent behavior and lack of functional impairment typically lead to delayed diagnosis; most patients present with metastatic disease [11].

The treatment of ASPS is based on its stage. Surgical excision could be curative for some patients in the early stages; nevertheless, long‐term recurrence and metastasis are not rare [12]. However, according to a study by Ogose et al., there was no local recurrence after wide excision or amputation following a mean follow‐up period of 70 months [13]. Surgical excision with clear margins is the cornerstone for managing localized and minimally metastatic ASPS. We successfully employed this approach, and no local recurrence was observed during our follow‐up.

Radiotherapy and conventional chemotherapy were not used to manage our patient. According to the literature, radiotherapy in ASPS might benefit brain metastasis. However, its effectiveness in pulmonary metastasis treatment has not been observed. A study assessing the brain metastases of different sarcomas, including ASPSs, reported an 88% improvement in the local control rate by concurrently using gamma knife stereotactic radiosurgery and 16 grays (Gy) [14]. However, one case report implied a potential benefit of radiotherapy for lung metastasis from ASPS only after using a much higher dose of 44.8 Gy [15]. The ineffectiveness of chemotherapeutic medications in treating ASPS has been well known since the late 20th century. Reichardt et al. stated that only 4% of patients experienced CR in the disease course using chemotherapeutic agents [2, 16].

Regarding the anti‐angiogenic effects of TKIs, they are increasingly being suggested as a valuable option in managing ASPS, particularly among patients presenting with metastatic disease [3, 17]. To date, pazopanib and sunitinib are approved for the treatment of ASPS by the Chinese Society of Clinical Oncologists (CSCO) and National Comprehensive Cancer Network (NCCN) guidelines [2]. Pazopanib, a multi‐targeted TKI with an inhibiting effect on vascular endothelial growth factor and platelet‐derived growth factor signaling, is prevalently associated with adverse drug effects such as GI side effects and fatigue, which are reported to be the leading cause of drug discontinuation according to the Pazopanib for metastatic soft‐tissue sarcoma (PALETTE) study. In this study, hair hypopigmentation occurred in 38% of patients, which was reported to be mild in almost all affected patients [17].

In our case, the severe side effects of pazopanib, including severe GI distress and complete hair depigmentation, led us to switch the patient's medications to immunotherapy despite achieving SD. At the time, selecting the best treatment option was a medical challenge. ICIs, including nivolumab, ipilimumab, and atezolizumab, have shown possible benefits in treating ASPS due to their activity against programmed cell death protein‐1 (PD‐1), cytotoxic T‐lymphocyte‐associated protein 4 (CTLA‐4), and programmed cell death protein‐ligand‐1 (PD‐L1), respectively. Among ICIs, only pembrolizumab had been approved by the CSCO and NCCN guidelines. In contrast, others had shown only limited benefits in treating ASPS in a few studies, especially in pediatric and adolescent subjects [9, 18]. Furthermore, atezolizumab has been shown to be effective in brain metastasis of ASPS despite a lack of radiologic evidence in a case report [19]. Additionally, combination therapy using two ICIs has proven to be more effective than monotherapy with a single ICI in advanced malignancies [20, 21]. The combination therapy discussed in previous studies has been shown to be effective when it includes an anti‐CTLA‐4 along with either an anti‐PD‐1 or anti‐PD‐L1 [22, 23]. The expression of these molecules in the tumor and its environment could stem from TFE3 upregulating genes in the transforming growth factor (TGF)–β pathway, leading to an inflammation that may enhance PD‐1 and PD‐L1 expression [24, 25]. The expression of tumor antigens PD‐1, PD‐L1, and CTLA‐4 in patients does not necessarily predict response to specific antigen inhibitor therapies or other treatment modalities, as highlighted in earlier studies on ASPS, non‐small cell lung cancer, melanoma, and renal cell carcinoma [24, 26]. Therefore, we chose the option that provided the broadest coverage and had the most significant supporting evidence from prior surveys: a combined regimen of ipilimumab and nivolumab [24, 27]. This combination has been shown to have relatively minimal side effects and good tolerability in the pediatric population. Its efficacy has been demonstrated to surpass that of single‐agent blockade of PD‐1 or PD‐L1. Monotherapy with anti‐PD‐1 or anti‐PD‐L1 is limited to treating Hodgkin and non‐Hodgkin lymphoma and cancers arising from biallelic mismatch repair. Furthermore, single‐agent blockade of CTLA‐4 has not shown any benefit in treating pediatric melanoma and solid tumors [28, 29]. Therefore, we decided to proceed with concurrent treatment using ipilimumab and nivolumab, which are approved for treating metastatic melanoma and hepatoblastoma [28]. This regimen was highlighted by Conley et al. in their report on a refractory ASPS in a 29‐year‐old man, which resulted in an SD [24]. In another report, this regimen resulted in a partial response in a 25‐year‐old man but was finally discontinued due to adverse effects [28]. In contrast to other reports, our patient achieved a CR after being treated with a combination regimen of ICIs. Additionally, in previous studies, the cause of switching from TKIs to novel therapeutic agents was treatment failure [9, 24]. However, in our patient, despite achieving an SD, the medications were changed due to the adverse effects of pazopanib. Finally, after treatment concurrently with ipilimumab and nivolumab, the treatment with nivolumab was continued for 1 year, and the patient did not experience any local or distant recurrence.

## 4 Conclusion

ASPS is a rare malignancy with high morbidity and mortality rates. While conventional chemotherapy is almost ineffective in treatment, novel treatments include TKIs and ICIs. Nivolumab and ipilimumab, an immunotherapy regimen with activity against PD‐1 and CTLA‐4, can be used in metastatic ASPS with negligible adverse effects and high efficacy. This report could provide pediatric oncologists with more options and evidence for treating ASPS. However, observational and clinical trial studies are needed to address this issue.

## Author Contributions

**A.E.:** conceptualized the case report. **A.S. and H.S.M.:** collected the data. **A.S. and M.O.:** participated in drafting the manuscript. **R.R.:** reviewed the draft and edited it. **A.E.:** revised the manuscript and provided expert comments. All authors have reviewed and approved the final manuscript and take full responsibility for their work.

## Ethics Statement

Verbal and written permission from the parents and the patient's verbal consent for publication were obtained.

## Consent

The authors have nothing to report.

## Conflicts of Interest

The authors declare no conflicts of interest.

## Data Availability

The data that support the findings of this study are available on request from the corresponding author. The data are not publicly available due to privacy or ethical restrictions.

## References

[1] P. Casali , N. Abecassis , S. Bauer , et al., “Soft Tissue and Visceral Sarcomas: ESMO–EURACAN Clinical Practice Guidelines for Diagnosis, Treatment and Follow‐Up,” Annals of Oncology 29 (2018): iv51–iv67. 29846498 10.1093/annonc/mdy096

[2] X. Chang , Y. Li , X. Xue , H. Zhou , and L. Hou , “The Current Management of Alveolar Soft Part Sarcomas,” Medicine 100, no. 31 (2021): e26805. 34397835 10.1097/MD.0000000000026805 PMC8341245

[3] T. Fujiwara , T. Kunisada , E. Nakata , et al., “Advances in Treatment of Alveolar Soft Part Sarcoma: An Updated Review,” Japanese Journal of Clinical Oncology 53, no. 11 (2023): 1009–1018. 37626447 10.1093/jjco/hyad102 PMC10632598

[4] A. Aksionau , N. E. Dela Cruz , A. T. Meram , H. Cuellar‐Saenz , J. R. Aveni , and H. Takei , “Lingual Alveolar Soft Part Sarcoma in a 78‐Year‐Old Woman: A Case Report and Comprehensive Review of the Literature From 1952 to 2022,” Head and Neck Pathology 17, no. 1 (2023): 265–274. 36303014 10.1007/s12105-022-01505-x PMC10063713

[5] R. J. Flores , D. J. Harrison , N. C. Federman , et al., “Alveolar Soft Part Sarcoma in Children and Young Adults: A Report of 69 Cases,” Pediatric Blood & Cancer 65, no. 5 (2018): e26953. 29350467 10.1002/pbc.26953

[6] S. Stacchiotti , O. Mir , A. Le Cesne , et al., “Activity of Pazopanib and Trabectedin in Advanced Alveolar Soft Part Sarcoma,” Oncologist 23, no. 1 (2018): 62–70. 28754721 10.1634/theoncologist.2017-0161 PMC5759809

[7] B. A. Wilky , M. M. Trucco , T. K. Subhawong , et al., “Axitinib Plus Pembrolizumab in Patients With Advanced Sarcomas Including Alveolar Soft‐Part Sarcoma: A Single‐Centre, Single‐Arm, Phase 2 Trial,” Lancet Oncology 20, no. 6 (2019): 837–848. 31078463 10.1016/S1470-2045(19)30153-6

[8] M. Anastasiou , A. Kyriazoglou , I. Kotsantis , et al., “Immune Checkpoint Inhibitors in Sarcomas: A Systematic Review,” Immuno‐Oncology and Technology 20 (2023): 100407. 10.1016/j.iotech.2023.100407 PMC10772240 38192615

[9] D. J. Kuo , J. S. Menell , and J. L. G. Bender , “Treatment of Metastatic, Refractory Alveolar Soft Part Sarcoma: Case Reports and Literature Review of Treatment Options in the Era of Targeted Therapy,” Journal of Pediatric Hematology/Oncology 38, no. 5 (2016): e169–e172. 27164526 10.1097/MPH.0000000000000571

[10] J. A. Park and N.‐K. V. Cheung , “Limitations and Opportunities for Immune Checkpoint Inhibitors in Pediatric Malignancies,” Cancer Treatment Reviews 58 (2017): 22–33. 28622628 10.1016/j.ctrv.2017.05.006 PMC5524462

[11] T. Fujiwara , E. Nakata , T. Kunisada , T. Ozaki , and A. Kawai , “Alveolar Soft Part Sarcoma: Progress Toward Improvement in Survival? A Population‐Based Study,” BMC Cancer 22, no. 1 (2022): 891. 35971085 10.1186/s12885-022-09968-5 PMC9377116

[12] L. Paoluzzi and R. G. Maki , “Diagnosis, Prognosis, and Treatment of Alveolar Soft‐Part Sarcoma: A Review,” JAMA Oncology 5, no. 2 (2019): 254–260. 30347044 10.1001/jamaoncol.2018.4490

[13] A. Ogose , Y. Yazawa , T. Ueda , et al., “Alveolar Soft Part Sarcoma in Japan: Multi‐Institutional Study of 57 Patients From the Japanese Musculoskeletal Oncology Group,” Oncology 65, no. 1 (2003): 7–13. 10.1159/000071199 12837977

[14] T. Flannery , H. Kano , A. Niranjan , et al., “Gamma Knife Radiosurgery as a Therapeutic Strategy for Intracranial Sarcomatous Metastases,” International Journal of Radiation Oncology, Biology, Physics 76, no. 2 (2010): 513–519. 19467792 10.1016/j.ijrobp.2009.02.007

[15] M. Hanzer , A. Nebl , S. Spendel , A. Pilhatsch , C. Urban , and M. Benesch , “Necrosis of a Skin Autograft After Short‐Term Treatment With Sunitinib in a 14‐Year‐Old Girl With Metastatic Alveolar Soft Part Sarcoma of the Thigh,” Klinische Pädiatrie 222, no. 3 (2010): 184–186. 20514624 10.1055/s-0030-1249093

[16] P. Reichardt , T. Lindner , D. Pink , P. Thuss‐Patience , A. Kretzschmar , and B. Dörken , “Chemotherapy in Alveolar Soft Part Sarcomas: What Do We Know?,” European Journal of Cancer 39, no. 11 (2003): 1511–1516. 12855256 10.1016/s0959-8049(03)00264-8

[17] W. T. Van Der Graaf , J.‐Y. Blay , S. P. Chawla , et al., “Pazopanib for Metastatic Soft‐Tissue Sarcoma (PALETTE): A Randomised, Double‐Blind, Placebo‐Controlled Phase 3 Trial,” Lancet 379, no. 9829 (2012): 1879–1886. 22595799 10.1016/S0140-6736(12)60651-5

[18] P. Aijaz , H. Sohail , M. A. Niazi , and A. Kamran , “Complete Response to Pembrolizumab in Stage IV Alveolar Soft Part Sarcoma After Failure of Four Lines of Treatment: A Case Report and Literature Review,” Cureus 16, no. 6 (2024): e62094. 38962626 10.7759/cureus.62094 PMC11221393

[19] T. A. Vander Jagt , L. E. Davis , M. D. Thakur , C. Franz , and J. M. Pollock , “Pseudoprogression of CNS Metastatic Disease of Alveolar Soft Part Sarcoma During Anti‐PDL1 Treatment,” Radiology Case Reports 13, no. 4 (2018): 882–885. 29991973 10.1016/j.radcr.2018.05.013 PMC6037874

[20] J. Larkin , V. Chiarion‐Sileni , R. Gonzalez , et al., “Combined Nivolumab and Ipilimumab or Monotherapy in Untreated Melanoma,” New England Journal of Medicine 373, no. 1 (2015): 23–34. 26027431 10.1056/NEJMoa1504030 PMC5698905

[21] X. Ma , Y. Zhang , S. Wang , H. Wei , and J. Yu , “Immune Checkpoint Inhibitor (ICI) Combination Therapy Compared to Monotherapy in Advanced Solid Cancer: A Systematic Review,” Journal of Cancer 12, no. 5 (2021): 1318–1333. 33531977 10.7150/jca.49174 PMC7847663

[22] A. R. Almutairi , A. McBride , M. Slack , B. L. Erstad , and I. Abraham , “Potential Immune‐Related Adverse Events Associated With Monotherapy and Combination Therapy of Ipilimumab, Nivolumab, and Pembrolizumab for Advanced Melanoma: A Systematic Review and Meta‐Analysis,” Frontiers in Oncology 10 (2020): 91. 32117745 10.3389/fonc.2020.00091 PMC7033582

[23] A. Gikandi , S. N. Chi , K. K. Yeo , et al., “Off‐Label Prescribing of Immune Checkpoint Inhibitor Therapy at a Single Pediatric Cancer Center,” Cancer Medicine 13, no. 8 (2024): e7154. 38629258 10.1002/cam4.7154 PMC11022150

[24] A. P. Conley , C. M. Zobniw , K. Posey , et al., “Positive Tumor Response to Combined Checkpoint Inhibitors in a Patient With Refractory Alveolar Soft Part Sarcoma: A Case Report,” Journal of Global Oncology 4 (2018): 1–6. 10.1200/JGO.2017.009993 PMC6180844 30241159

[25] M. A. Travis and D. Sheppard , “TGF‐β Activation and Function in Immunity,” Annual Review of Immunology 32, no. 1 (2014): 51–82. 10.1146/annurev-immunol-032713-120257 PMC4010192 24313777

[26] M. K. Callahan , M. A. Postow , and J. D. Wolchok , “CTLA‐4 and PD‐1 Pathway Blockade: Combinations in the Clinic,” Frontiers in Oncology 4 (2015): 385. 25642417 10.3389/fonc.2014.00385 PMC4295550

[27] J. Lewin , S. Davidson , N. D. Anderson , et al., “Response to Immune Checkpoint Inhibition in Two Patients With Alveolar Soft‐Part Sarcoma,” Cancer Immunology Research 6, no. 9 (2018): 1001–1007. 30018044 10.1158/2326-6066.CIR-18-0037

[28] K. L. Davis , E. Fox , E. Isikwei , et al., “A Phase I/II Trial of Nivolumab Plus Ipilimumab in Children and Young Adults With Relapsed/Refractory Solid Tumors: A Children's Oncology Group Study ADVL1412,” Clinical Cancer Research 28, no. 23 (2022): 5088–5097. 36190525 10.1158/1078-0432.CCR-22-2164 PMC10597535

[29] B. Geoerger , H. J. Kang , M. Yalon‐Oren , et al., “Pembrolizumab in Paediatric Patients With Advanced Melanoma or a PD‐L1‐Positive, Advanced, Relapsed, or Refractory Solid Tumour or Lymphoma (KEYNOTE‐051): Interim Analysis of an Open‐Label, Single‐Arm, Phase 1–2 Trial,” Lancet Oncology 21, no. 1 (2020): 121–133. 31812554 10.1016/S1470-2045(19)30671-0

